# Supplementary material for: Reconciling Mining with the Conservation of Cave Biodiversity: A Quantitative Baseline to Help Establish Conservation Priorities
Source: PLoS One. 2016 Dec 20;11(12):e0168348. doi: 10.1371/journal.pone.0168348 (PMC5173368; doi:10.1371/journal.pone.0168348)
Supplement: S1 Dataset — (ZIP) [file pone.0168348.s002.zip › Taxa/Serra Norte/SN_2013/N5S/Lista N5S-60.pdf]

CAVIDADE N5S-0060

Classe	Ordem	Fam/Outros	Gên/Outros	Espécie	S-U	U-S	Zon.
Arachnida	Acari	Opilioacaridae		sp.1		X	E
Arachnida	Amblypygi	Charinidae	*Charinus*	sp.1		5	E
Arachnida	Amblypygi	Phrynidae	*Heterophrynus*	*longicornis*		1	E
Arachnida	Araneae	Corinnidae		jovem		1	E
Arachnida	Araneae	Filistatidae		jovem	X		E
Arachnida	Araneae	Ochyroceratidae		jovem		X	E
Arachnida	Araneae	Pholcidae	*Mesabolivar*	*aurantiacus*		X	E
Arachnida	Araneae	Pholcidae	*Mesabolivar*	sp.1		X	E
Arachnida	Araneae	Salticidae		sp.2		X	E
Arachnida	Araneae	Tetragnathidae		jovem	X		E
Arachnida	Araneae	Theraphosidae	aff. *Holothele*	sp.1		1	E
Arachnida	Araneae	Theridiidae		jovem	X		E
Arachnida	Araneae	Theridiosomatidae		jovem		X	E
Arachnida	Opiliones	Cosmetidae	*Roquettea*	*singularis*		1	E
Arachnida	Pseudoscorpiones	Chthoniidae		jovem		X	E
Arachnida	Pseudoscorpiones	Olpiidae		sp.1	X		E
Entognatha	Collembola	Entomobryidae		sp.2	X		E
Entognatha	Collembola	Paronellidae		sp.1		X	E
Entognatha	Diplura	Campodeidae		sp.1		X	E
Insecta	Blattodea	Blattellidae		sp.1	3		E
Insecta	Coleoptera	Carabidae		sp.3		X	E
Insecta	Coleoptera	Curculionidae	Scolytinae	sp.2	X		E
Insecta	Diptera	Cecidomyiidae	Cecidomyiinae	sp.	X	X	E
Insecta	Diptera	Dolichopodidae		sp.	X		E
Insecta	Diptera	Ephydridae		sp.		X	E
Insecta	Heteroptera	Reduviidae	Reduviinae	sp.	2	2	E
Insecta	Homoptera	Cixiidae		sp.3	X		E
Insecta	Hymenoptera	Formicidae	*Camponotus*	sp.1	X		E
Insecta	Hymenoptera	Formicidae	*Camponotus*	sp.2		X	E
Insecta	Hymenoptera	Formicidae	*Cephalotes*	sp.1	X		E
Insecta	Hymenoptera	Formicidae	*Crematogaster*	sp.1	X		E
Insecta	Hymenoptera	Formicidae	*Dolichoderus*	aff. *bispinosus*		X	E
Insecta	Hymenoptera	Formicidae	*Pachycondyla*	sp.2		X	E
Insecta	Hymenoptera	Formicidae	*Paratrechina*	sp.1		X	E
Insecta	Hymenoptera	Formicidae	*Pheidole*	sp.2	X		E
Insecta	Isoptera	Termitidae	*Microcerotermes*	sp.		X	E
Insecta	Isoptera	Termitidae	*Nasutitermes*	sp.	X	X	E
Insecta	Lepidoptera	Noctuoidea		sp.6		2	E
Insecta	Lepidoptera	Tineoidea		sp.3		X	E
Insecta	Orthoptera	Phalangopsidae	*Paraclodes*	sp.	1		E
Insecta	Psocoptera	Ptiloneuridae	*Triplocania*	sp.9	X		E
Insecta	Thysanura	Nicoletiidae		sp.1		X	E
Gastropoda	Pulmonata	Subulinidae		sp.1		X	E
Gastropoda	Pulmonata	Subulinidae		sp.5	X		E
Turbellaria				sp.1		X	E
Mammalia	Chiroptera	Emballonuridae	*Peropteryx*	*kappleri*		3	E
Reptilia	Squamata	Colubridae		sp.	1		E
